# Supplementary material for: Abemaciclib is a potent inhibitor of DYRK1A and HIP kinases involved in transcriptional regulation
Source: Nat Commun. 2021 Nov 16;12:6607. doi: 10.1038/s41467-021-26935-z (PMC8595372; doi:10.1038/s41467-021-26935-z)
Supplement: Supplementary file 1 — Supplementary Information [file 41467_2021_26935_MOESM1_ESM.pdf]

Supplementary Information

Abemaciclib is a potent inhibitor of DYRK1A and HIP kinases involved in transcriptional regulation

Ines H. Kaltheuner[1], Kanchan Anand[1], Jonas Moecking[1], Robert Düster[1], Jinhua Wang[2,3], Nathanael S. Gray[4], and Matthias Geyer[1,*]

[1] Institute of Structural Biology, University of Bonn, Venusberg-Campus 1, 53127 Bonn, Germany.
[2] Department of Cancer Biology, Dana-Farber Cancer Institute, Boston, Massachusetts 02215, USA.
[3] Department of Biological Chemistry and Molecular Pharmacology, Harvard Medical School, Boston, Massachusetts 02215, USA.
[4] Department of Chemical and Systems Biology, Chem-H and the Stanford Cancer Institute, Stanford School of Medicine, Stanford University, Stanford, California, 94305, USA.

* Correspondence should be addressed to: matthias.geyer@uni-bonn.de

The PDF file includes:

- Supplementary Figures S1 – S6
- Supplementary Tables 1 – 3

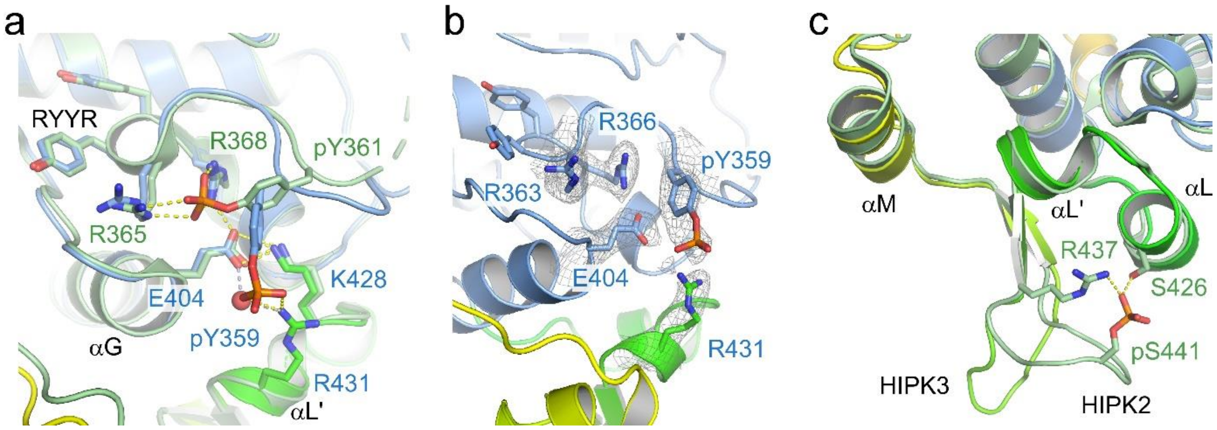

Supplementary Fig. 1 Coordination of the phosphorylated tyrosine and the CMGC insert region in HIPK2 and HIPK3.
a Overlay of the conserved phospho-tyrosine in HIPK3 (blue) and HIPK2 (light green; PDB code: 6P5S). Electrostatic and hydrogen bond interactions between neighboring residues are indicated. **b** Close up of the pY359 coordination in the HIPK3 activation segment. The final 2 F_o-F_c density is displayed at 1σ. **c** Overlay of the CMGC insert in HIPK3 (blue) and HIPK2 (light green, 6P5S). In HIPK2, pS441 makes contacts with residues from the αL-helix whereas no such phosphorylation is present in HIPK3.

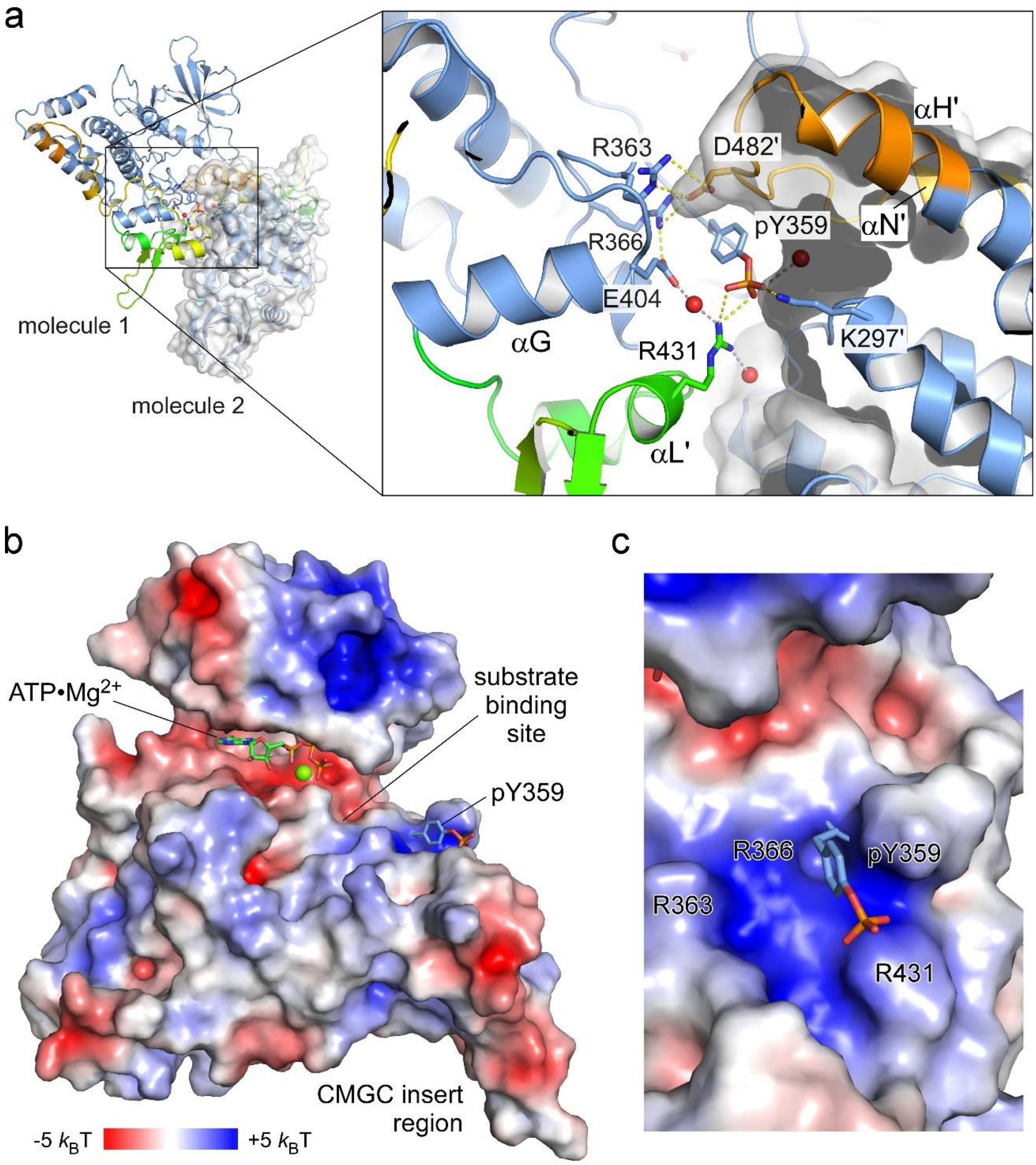

Supplementary Fig. 2 Crystallographic assembly and surface charge of HIPK3.
a In the apo-HIPK3 structure, pY359 of the activation loop forms a salt bridge with R431 (cartoon representation) and a weak electrostatic interaction with K297' of a symmetry related molecule (surface representation). The carboxy side chain of D482' from the same symmetry related molecule sticks in between the R_{363}YYR motif by forming tight salt bridges. **b** Display of the electrostatic surface potential of HIPK3. The ATP•Mg^{2+} nucleotide assembly was modeled into the apo HIPK3 structure from a superimposition with the Cdk2•ATP•Mg^{2+} crystal structure (1QMZ). The basic patch in the N-lobe (top) is formed amongst others by R205 and K229. **c** Close up of the surface potential surrounding pY359. A basic surface potential is delineated by R363, R366 and R431. Electrostatic surface charge is shown from -5 k_BT (red) to +5 k_BT (blue).

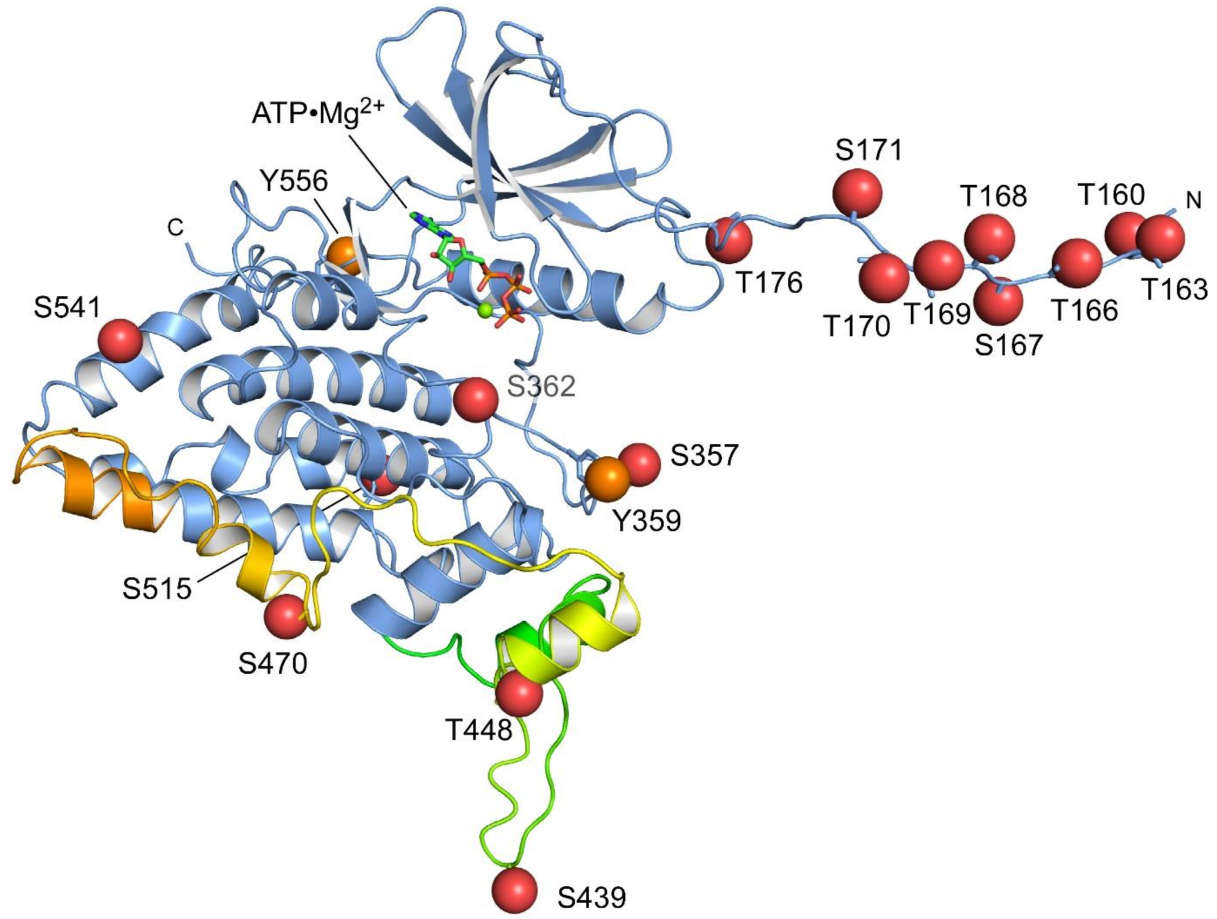

Supplementary Fig. 3 Phosphorylation sites in HIP kinases mapped onto HIPK3.
Model of the HIPK3 construct (159-562) used in this study based on a fusion of the human HIPK3 apo structure determined here (7O7I) from residues 193-546 with the AlphaFold model (AF-Q9H422-F1-model_v1) for residues 159-192 and 547-562. All positions of phosphorylated residues in HIP kinases 1 to 4 found by mass spectrometry analysis (shown in Fig. 2 and listed in Supplementary Table 2) were projected onto this model of HIPK3. Phosphorylation sites are shown by red (Ser/Thr) and orange (Tyr) spheres, respectively, using the amino acid numbering of HIPK3. All phospho-sites were found to be on the surface of the structure supporting their accessibility for modification. A bound ATP•Mg^{2+} nucleotide-ion substrate in the ATP-binding site superimposed from the structure of Cdk2/CycA (1QMZ)[19] is shown for clarity.

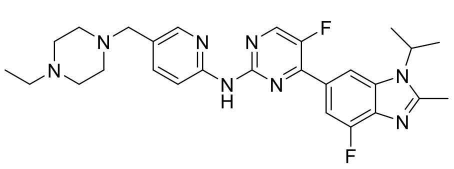

abemaciclib

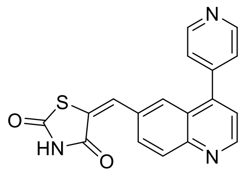

GSK1059615

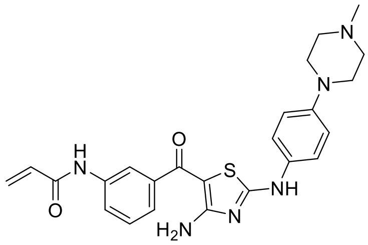

JWD-065

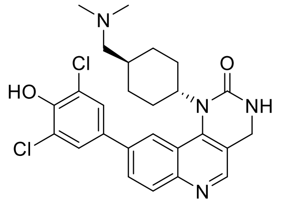

HTH-01-091

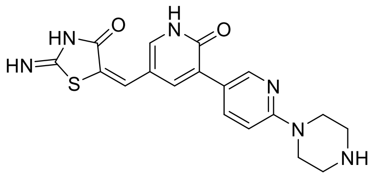

CVM-06-033-2

Supplementary Fig. 4 Chemical structures of top five selected drugs against HIPK3. In a kinase assay using recombinant protein the five compounds abemaciclib, GSK1059615, JWD-065, HTH-01-091, and CVM-06-033-2 reduced HIPK3 activity most potently.

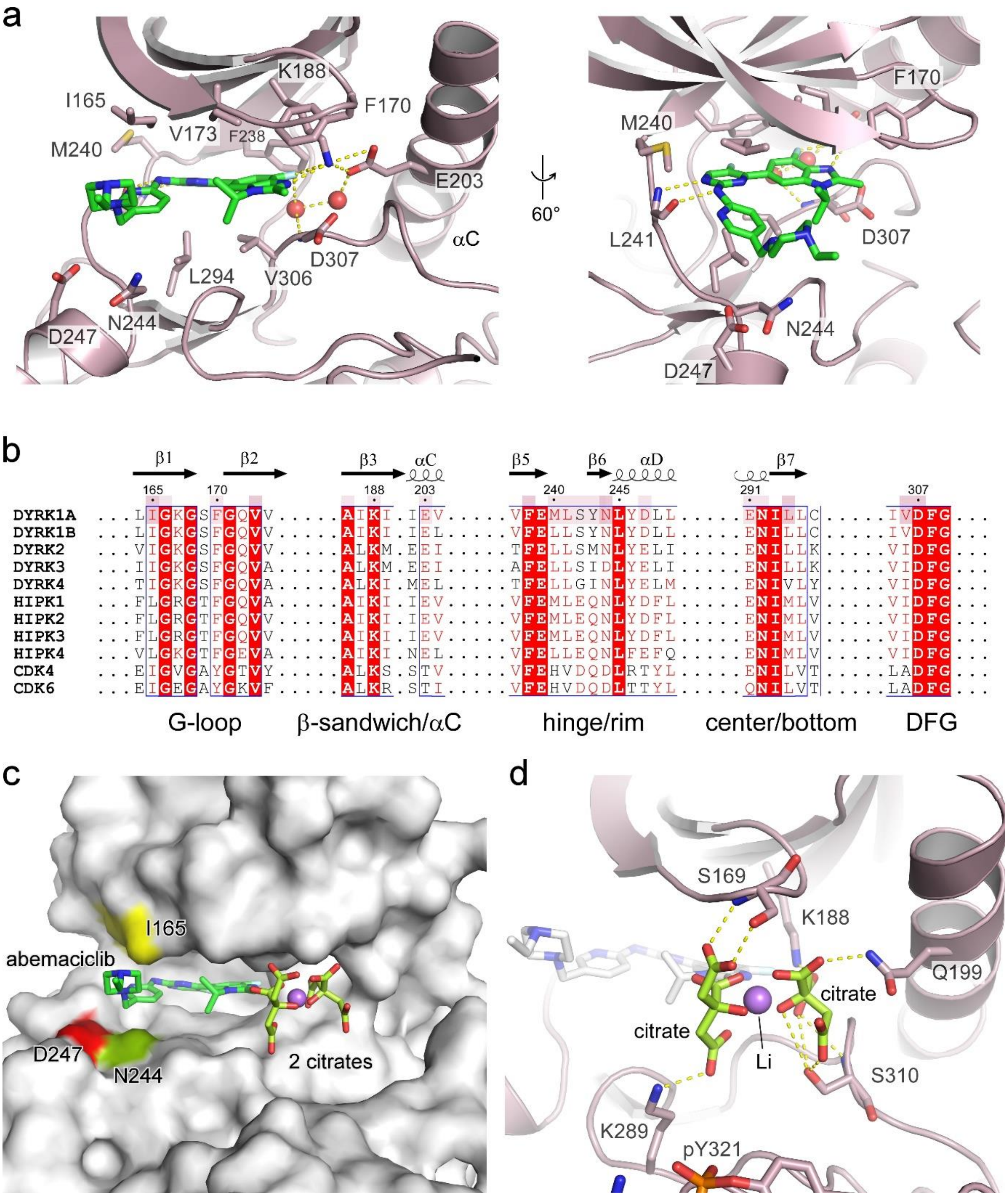

Supplementary Fig. 5 Crystal structure of DYRK1A in complex with abemaciclib.
a Crystal Structure of DYRK1A bound to abemaciclib (PDB code 7O7K). Interacting residues as well as water mediated contacts are labelled. The central 2-aminopyrimidine group of abemaciclib interacts with the hinge region of DYRK1A (right). **b** Sequence alignment of human DYRK and HIPK kinases as well as Cdk4 and Cdk6 in the interacting regions with abemaciclib. Residues in DYRK1A involved in direct interactions with abemaciclib are boxed in the top line (with more than 8 Å^2 buried surface area in light rose and more than 20 Å^2 in dark rose, respectively). **c** In the structure of DYRK1A two citrate molecules (lime) are found at the ATP exit site where the catalytic reaction with the kinase substrate takes place. **d** The two citrate molecules are heavily hydrogen-bonded to S310 and S169 in DYRK1A as well as K188, Q199 and K289.

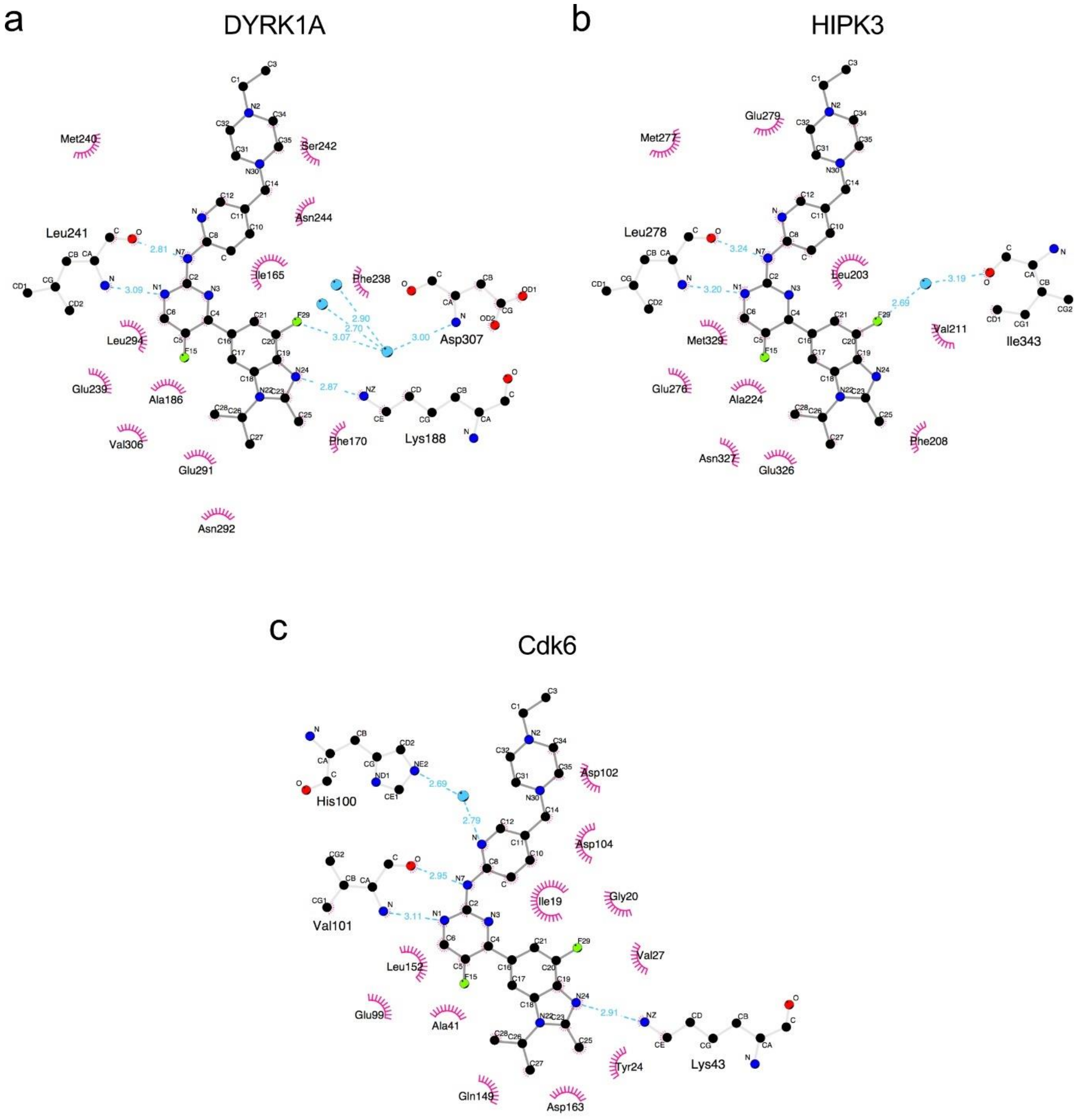

Supplementary Fig. 6 Interactions of CMGC kinases DYRK1A, HIPK3 and Cdk6 with abemaciclib.

Lig-plot of the abemaciclib-binding interfaces. **a** DYRK1A (PDB 7O7K). **b** HIPK3 (PDB 7O7J). **c** Cdk6 (PDB 5L2S). Direct and water-mediated hydrogen bonds to the drug are indicated. Hydrophobic and van-der-Waals interactions are shown by red circles around the contributing residues.

Supplementary Table 1: Crystallographic data collection and refinement statistics.

	apo-HIPK3	HIPK3–abemaciclib	DYRK1A–abemaciclib
Data collection [a]			
Beam line	SLS X06SA	SLS X06SA	SLS X06SA
Wavelength [Å]	0.9999	0.9999	1.0000
Space group	$P\ 3_2\ 2\ 1$	$P\ 3_2\ 2\ 1$	$P\ 2_1\ 2_1\ 2_1$
Unit cell: a, b, c [Å]<br>α, β, γ [°]	80.28, 80.28, 181.99<br>90, 90, 120	81.06, 81.06, 178.82<br>90, 90, 120	76.70, 109.98 112.49<br>90, 90, 90
Resolution range [Å]	45.71–2.50 (2.59–2.50)	45.44–2.81 (2.91–2.81)	41.93–1.82 (1.885–1.82)
Unique reflections	17,854 (236)	17,230 (1679)	85,615 (8,460)
Multiplicity	17.0 (10.5)	16.3 (17.4)	13.4 (13.6)
Completeness spherical (%)<br>Completeness ellipsoidal (%)	73.69 (9.84)<br>93.8	99.91 (100.00)	99.59 (99.35)
Mean I/sigma(I)	12.38 (0.85)	23.68 (1.79)	16.36 (1.41)
R_{meas}	0.126 (5.02)	0.06367 (1.596)	0.07891 (1.718)
$CC_{1/2}$	0.89 (0.38)	0.999 (0.825)	0.999 (0.836)
Reflections used in refinement	17,854 (236)	17,222 (1,679)	85,539 (8,447)
Reflections used for R-free	938 (16)	862 (84)	4,279 (422)
Refinement			
Model content	A: HIPK3 (184–550)	A: HIPK3(184–551): abemaciclib	A: DYRK1A (134-480,∆411-412): abemaciclib, 2 citrates, 1 Li<br>B: DYRK1B (133-480,∆407-411): abemaciclib, 2 citrates, 1 Li
# of atoms macromolecules	2932	2940	5688
# of ligands	4	69	224
# of solvent	51	15	375
Solvent content [%]	49	64	57
R_{work}	0.2429 (0.5255)	0.2464 (0.3327)	0.1856 (0.3321)
R_{free}	0.2730 (0.5908)	0.2748 (0.3842)	0.2111 (0.3434)
RMS deviations bonds [Å]	0.002	0.007	0.009
RMS deviations angles [°]	0.44	1.25	1.09
Ramachandran favored (%)	95.86	92.29	96.44
Ramachandran allowed (%)	3.87	6.61	3.41
Average B-factor	81.57	115.63	48.45
Macromolecules	81.61	115.86	48.08
ligands	84.80	98.03	50.98
solvent	78.93	113.47	52.58
PDB accession code	7O7I	7O7J	7O7K

[a] Values in parentheses are for the highest resolution shell.
R_{free}-value is equivalent to the R-value but is calculated for 5% of the reflections chosen at random and omitted from the refinement process.

Supplementary Table 2: Identification of phospho-sites in HIPKs and DYRK1A by peptide finger print mass spectrometry.

Phospho-site	# of total peptides	# of phospho-peptides	% of phospho-peptides
**HIPK1 from *Sf9*** (total coverage 87%)			
**pY352**	28	18	64%
**HIPK2 from *E. coli*** (total coverage 89%)			
pT162	38	10	26%
pT165	38	20	53%
pT168	38	7	18%
pS169	38	8	21%
pT170	38	6	16%
pT172	38	13	34%
**pY361**	49	35	71%
pS364	49	2	4%
pS441	33	26	79%
pT450	91	37	41%
pY558	50	15	30%
**HIPK2 from *Sf9*** (total coverage 72%)			
**pY361**	19	10	53%
**HIPK3 from *E. coli*** (total coverage 87%)			
pT163	33	10	30%
pT169	33	10	30%
pS171	33	6	18%
**pY359**	26	10	39%
pT515	52	8	15%
**HIPK3 from *Sf9*** (total coverage 89%)			
pT176	24	8	33%
pS357	102	12	12%
**pY359**	173	122	71%
pT515	95	36	38%
pS541	11	4	36%
**MBP-HIPK4 from *Sf9*** (total coverage 81%)			
**pY175**	32	13	41%
pS178	20	3	15%
pS287	12	3	25%
pS337	41	8	20%
pS406	52	12	23%
pS411	52	14	27%
pS511	5	4	80%
**DYRK1A from *E. coli*** (total coverage 76%)			
pY136	35	7	20%
**pY321**	63	57	91%

Supplementary Table 3: Thermal stability of DYRK1A, HIPKs and selected CDKs upon addition of abemaciclib.

Protein[a]	Treatment	T_m (°C)	ΔT_m to control (°C)
DYRK1A	buffer	56.9	
	1 µM ADP	56.8	-0.1
	10 µM ADP	56.9	0.0
	100 µM ADP	56.8	-0.1
DYRK1A	buffer	56.9	
	1 µM ATP	56.7	-0.2
	10 µM ATP	57.0	+0.1
	100 µM ATP	56.9	0.0
DYRK1A	DMSO	54.7	
	1 µM abemaciclib	55.3	+0.6
	10 µM abemaciclib	57.8	+3.1
	100 µM abemaciclib	59.9	+5.2
HIPK1	DMSO	49.5	
	1 µM abemaciclib	49.9	+0.4
	10 µM abemaciclib	55.9	+6.4
	100 µM abemaciclib	58.2	+8.7
HIPK2	DMSO	52.1	
	1 µM abemaciclib	53.6	+1.5
	10 µM abemaciclib	56.8	+4.7
	100 µM abemaciclib	59.3	+7.2
HIPK3	DMSO	45.3	
	1 µM abemaciclib	45.5	+0.2
	10 µM abemaciclib	50.6	+5.3
	100 µM abemaciclib	52.7	+7.4
MBP-HIPK4	DMSO	47.9	
	1 µM abemaciclib	48.5	+0.6
	10 µM abemaciclib	49.2	+1.3
	100 µM abemaciclib	49.8	+1.9
Cdk4/CycD3	DMSO	55.2	
	1 µM abemaciclib	59.3	+4.1
	10 µM abemaciclib	61.0	+4.8
	100 µM abemaciclib	63.1	+7.9
Cdk9/CycT1	DMSO	52.0	
	1 µM abemaciclib	52.6	+0.6
	10 µM abemaciclib	54.4	+2.4
	100 µM abemaciclib	55.3	+3.3
MBP	DMSO	54.4	
	1 µM abemaciclib	54.4	0.0
	10 µM abemaciclib	54.5	+0.1
	100 µM abemaciclib	54.5	+0.1

[a] All proteins were used at a concentration of 5 µM. A total of 10% DMSO was used in the control and the abemaciclib samples. The melting temperatures (T_m) were determined with the nanoDSF technique using a Prometheus (NanoTemper) device.
