# Supplementary material for: Abemaciclib is a potent inhibitor of DYRK1A and HIP kinases involved in transcriptional regulation
Source: Nat Commun. 2021 Nov 16;12:6607. doi: 10.1038/s41467-021-26935-z (PMC8595372; doi:10.1038/s41467-021-26935-z)
Supplement: Supplementary file 3 — Reporting Summary [file 41467_2021_26935_MOESM3_ESM.pdf]

nature research

Corresponding author(s): Matthias Geyer

Last updated by author(s): Oct 25, 2021

Reporting Summary

Nature Research wishes to improve the reproducibility of the work that we publish. This form provides structure for consistency and transparency in reporting. For further information on Nature Research policies, see our Editorial Policies and the Editorial Policy Checklist.

Statistics

For all statistical analyses, confirm that the following items are present in the figure legend, table legend, main text, or Methods section.

n/a	Confirmed	
☐	☒	The exact sample size (*n*) for each experimental group/condition, given as a discrete number and unit of measurement
☐	☒	A statement on whether measurements were taken from distinct samples or whether the same sample was measured repeatedly
☒	☐	The statistical test(s) used AND whether they are one- or two-sided<br>*Only common tests should be described solely by name; describe more complex techniques in the Methods section.*
☒	☐	A description of all covariates tested
☒	☐	A description of any assumptions or corrections, such as tests of normality and adjustment for multiple comparisons
☐	☒	A full description of the statistical parameters including central tendency (e.g. means) or other basic estimates (e.g. regression coefficient) AND variation (e.g. standard deviation) or associated estimates of uncertainty (e.g. confidence intervals)
☒	☐	For null hypothesis testing, the test statistic (e.g. *F*, *t*, *r*) with confidence intervals, effect sizes, degrees of freedom and *P* value noted<br>*Give P values as exact values whenever suitable.*
☒	☐	For Bayesian analysis, information on the choice of priors and Markov chain Monte Carlo settings
☒	☐	For hierarchical and complex designs, identification of the appropriate level for tests and full reporting of outcomes
☒	☐	Estimates of effect sizes (e.g. Cohen's *d*, Pearson's *r*), indicating how they were calculated

Our web collection on statistics for biologists contains articles on many of the points above.

Software and code

Policy information about availability of computer code

Data collection	Diffraction data were collected at PX1 synchrotron beamline at Swiss Light Source, Villigen, Switzerland, equipped with an Eiger detector.
Data analysis	GraphPad Prism (v. 7), XDS program package (Feb. 5, 2021), StarAniso server (v.3.336), PHASER (v.2.8.3), Phenix (v.1.19.2-4158-000), Coot (v.0.9.4.1), PyMOL (v.2.4), Nanothemper Prometheus NT.48 PR.ThermControl (v.2.1.6), Cytiva Biacore Insight Evaluation Software (v.3.0.12.15655), BioRad ChemiDoc XRS+ system ImageLab (v.6.0.1), Scaffold4 (v.4.8.7)

For manuscripts utilizing custom algorithms or software that are central to the research but not yet described in published literature, software must be made available to editors and reviewers. We strongly encourage code deposition in a community repository (e.g. GitHub). See the Nature Research guidelines for submitting code & software for further information.

Data

Policy information about availability of data

All manuscripts must include a data availability statement. This statement should provide the following information, where applicable:

- Accession codes, unique identifiers, or web links for publicly available datasets
- A list of figures that have associated raw data
- A description of any restrictions on data availability

Uniprot accession codes from all proteins used in this study are: HIPK1 (Q86Z02), HIPK2 (Q9H2X6), HIPK3 (Q9H422), HIPK4 (Q3V016), DYRK1A (Q13627), Cdk4 (P11802), Cyclin D3 (P30281) Cdk9 (P50750), Cyclin T1 (O60563), Cdk12 (Q9NYV4), Cyclin K (O75909), CAK1 (P43568), Rpb1 (P24928), Rb1 (P06400), Spt5 (O00267), c-Myc (P01106)
Structure coordinates and diffraction data of the HIPK3 kinase domain in the apo form, the HIPK3-abemaciclib complex, and the DYRK1A-abemaciclib complex were deposited in the Protein Data Bank (http://www.pdb.org) under accession codes 7O7I, 7O7J, and 7O7K

April 2020

Field-specific reporting

Please select the one below that is the best fit for your research. If you are not sure, read the appropriate sections before making your selection.

☒ Life sciences ☐ Behavioural & social sciences ☐ Ecological, evolutionary & environmental sciences

For a reference copy of the document with all sections, see nature.com/documents/nr-reporting-summary-flat.pdf

Life sciences study design

All studies must disclose on these points even when the disclosure is negative.

Sample size	No experiments involving biological specimens were examined such that sample size does not apply. Therefore, sample size was not pre-determined using statistical methods.
Data exclusions	No data was excluded for analysis.
Replication	All biochemical experiments were performed independently and reproduced at least two times. All attempts of replication were successful.
Randomization	No animal or behavioral experiments were carried out in this study. Randomization was thus not necessary and was not performed in this study. All experiments were performed with appropriate internal negative and/or postitive controls as indicated.
Blinding	Blinding was not necessary for this study, since no animal or behavioral experiments were performed. All experiments were carried out with appropriate internal negative and/or postitive controls as indicated. Most results were validated by alternative techniques as described in the manuscript.

Reporting for specific materials, systems and methods

We require information from authors about some types of materials, experimental systems and methods used in many studies. Here, indicate whether each material, system or method listed is relevant to your study. If you are not sure if a list item applies to your research, read the appropriate section before selecting a response.

Materials & experimental systems

n/a	Involved in the study
☐	☒ Antibodies
☐	☒ Eukaryotic cell lines
☒	☐ Palaeontology and archaeology
☒	☐ Animals and other organisms
☒	☐ Human research participants
☒	☐ Clinical data
☒	☐ Dual use research of concern

Methods

n/a	Involved in the study
☒	☐ ChIP-seq
☒	☐ Flow cytometry
☒	☐ MRI-based neuroimaging

Antibodies

Antibodies used	All antibodies were purchased from the source cited in the manuscript. Dilution, source and catalog number are: Tyr1P (1:100, clone 3D12), Ser2P (1:100, clone 3E10), Ser5P (1:100, clone 3E8), Ser7P (1:100, clone 4E12) and Thr4P (1:100, clone G07) were a kind gift from Dirk Eick (Helmholtz Zentrum Munich, Germany) and were supplied as hybridoma supernatant. Secondary antibody chicken anti-rat IgG, HRP-coupled was purchased from Santa Cruz (1:5.000, sc-2964, #J0509).
Validation	All monoclonal antibodies were described previously (Chapman et al., 2007; Hintermair et al., 2012; Mayer et al. 2012).

Eukaryotic cell lines

Policy information about cell lines

Cell line source(s)	Sf9 insect cells: ThermoFisher, Catalogue no. 12659017, cell culture media: Sf-900TM III SFM
Authentication	Sf9 insect cell line was verified by manufacturer´s website and cellular identity was regularly checked by morphology. (ThermoFisher) https://www.thermofisher.com/order/catalog/product/12659017
Mycoplasma contamination	Mycoplasma test is not required for used cell line.
Commonly misidentified lines (See ICLAC register)	No commonly misidentified cell lines were used in this study.
