# Supplementary material for: Abemaciclib is a potent inhibitor of DYRK1A and HIP kinases involved in transcriptional regulation
Source: Nat Commun. 2021 Nov 16;12:6607. doi: 10.1038/s41467-021-26935-z (PMC8595372; doi:10.1038/s41467-021-26935-z)

Source Data Fig. 5c | Uncropped Gels

HIPK1

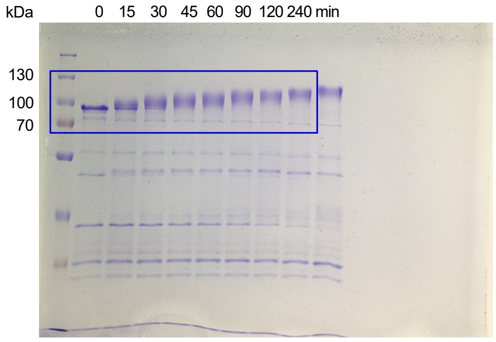

Molecular weight standard:
Pageruler Plus Prestained Protein Ladder.
10 to 25 kDa Marker (ThermoScientific #26620)

Blue boxes indicate parts of the blot displayed in the figure.

The last lane corresponds to overnight incubation.

HIPK2

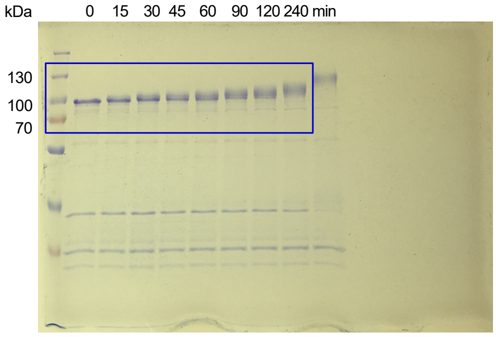

HIPK3

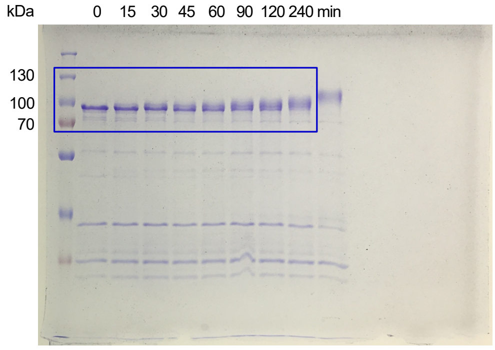

MBP-HIPK4

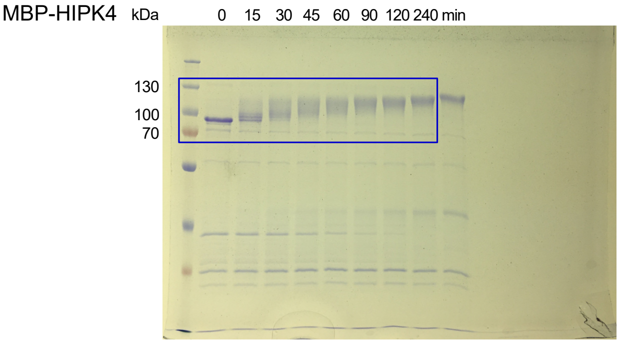

DYRK1A

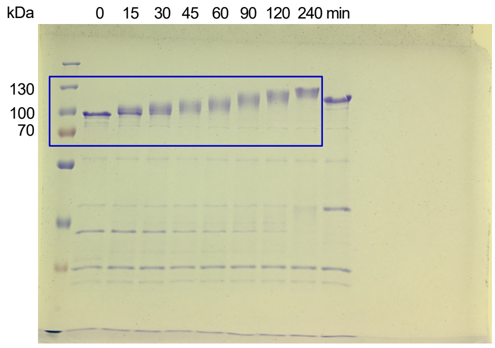

Supplement: Supplementary file 4 — Source Data [file 41467_2021_26935_MOESM4_ESM.zip › Source Data Fig.5c.pdf]
